# The Oligomeric Assemblies of Cytomegalovirus Core Nuclear Egress Proteins Are Associated with Host Kinases and Show Sensitivity to Antiviral Kinase Inhibitors

**DOI:** 10.3390/v14051021

**Published:** 2022-05-11

**Authors:** Jintawee Kicuntod, Sigrun Häge, Friedrich Hahn, Heinrich Sticht, Manfred Marschall

**Affiliations:** 1 Institute for Clinical and Molecular Virology, Friedrich-Alexander-Universität Erlangen-Nürnberg (FAU), 91054 Erlangen, Germany; jintawee.kicuntod@extern.uk-erlangen.de (J.K.); sigrun.haege@fau.de (S.H.); friedrich.hahn@uk-erlangen.de (F.H.); 2 Bioinformatics, Institute of Biochemistry, Friedrich-Alexander-Universität Erlangen-Nürnberg (FAU), 91054 Erlangen, Germany; heinrich.sticht@fau.de

**Keywords:** human cytomegalovirus, viral nucleocytoplasmic egress, nuclear egress complex (NEC), core NEC pUL50-pUL53, oligomerization, cross-linking, associated protein kinases, cyclin-dependent kinases (CDKs), kinase inhibitors, NEC-inhibitory potential, NEC as an antiviral target

## Abstract

The nucleo-cytoplasmic capsid egress of herpesviruses is a unique regulated process that ensures the efficiency of viral replication and release. For human cytomegalovirus (HCMV), the core of the nuclear egress complex (NEC) consists of the pUL50–pUL53 heterodimer that is able to oligomerize and thus to build hexameric lattices. These structures determine capsid binding and multicomponent protein interaction including NEC-associated host factors. The underlying characteristic of the core NEC formation is based on the N-terminal hook structure of pUL53 that binds into an alpha-helical groove of pUL50, and is thus described as a hook-into-groove interaction. This central regulatory element has recently been validated as a target of antiviral strategies, and first NEC-targeted prototypes of inhibitory small molecules were reported by our previous study. Here, we further analyzed the oligomerization properties of the viral NEC through an approach of chemical protein cross-linking. Findings were as follows: (i) a cross-link approach demonstrated the oligomeric state of the HCMV core NEC using material from HCMV-infected or plasmid-transfected cells, (ii) a Western blot-based identification of NEC-associated kinases using the cross-linked multicomponent NECs was successful, and (iii) we demonstrated the NEC-inhibitory and antiviral activity of specific inhibitors directed to these target kinases. Combined, the results strongly underline the functional importance of the oligomerization of the HCMV-specific NEC that is both phosphorylation-dependent and sensitive to antiviral kinase inhibitors.

## 1. Introduction

Human cytomegalovirus (HCMV) is a member of the family *Herpesviridae*, subfamily *Betaherpesvirinae*, and represents a major human pathogen with worldwide distribution. In immunocompetent individuals, HCMV infection mostly remains clinically mild. However, the infection of immunocompromised, immunosuppressed or immunonaïve hosts can lead to severe systemic diseases and life-threatening complications. Most importantly, HCMV infection of pregnant women that is associated with the congenital transmission of infection in approximately 33% of cases (cCMV) has been underestimated for decades. Today, cCMV raises a major clinical concern, as it represents the most frequent cause of non-genetic congenital abnormalities and spontaneous abortions. It spans a broad range of symptoms, including hearing loss, mental retardation or microcephaly, and leads to high morbidity as well as mortality in the unborn and infants. The severity of HCMV pathogenesis is generally determined by distinct factors of virus–host interaction, viral productivity, viremia, immune control and tissue tropism [1,2,3]. Regulation of the viral replication cycle is mediated by the complex interplay between viral and host proteins in a multifaceted way, including the formation of viral-cellular multiprotein complexes. In particular, the multicomponent HCMV-specific nuclear egress complex (NEC) exerts a rate-limiting step of productive replication and recently attracted high interest as it represents a putative target for novel antiviral strategies. Notably, genomic replication of HCMV occurs in the host cell nucleus followed by capsid assembly, encapsidation and cytoplasmic release of particles for further maturation. Thus, HCMV nucleocytoplasmic egress is a crucial step of the entire viral replication cycle, which depends on the correct and efficient formation of the viral NEC protein complex. The NEC assembly and functionality is primarily determined by two important viral egress proteins pUL50 and pUL53. These form a regulatory heterodimer that serves as a core NEC for the recruitment of associated effector proteins and viral capsids. In the case of HCMV, the recruited viral and cellular NEC-specific effectors have been identified through proteomic experimental settings, thus defining the multicomponent NEC [4,5]. The main regulators so far identified comprise the HCMV-encoded protein kinase pUL97, the multi-ligand binding protein p32/gC1qR, emerin, cellular kinases such as protein kinase C (PKC) and cyclin-dependent kinase 1 (CDK1), the prolyl cis/trans-isomerase Pin1 as well as a number of additional proteins [4,6]. Specifically, the kinases of the multicomponent NEC stimulate a phosphorylation-triggered disruption of the nuclear lamina that facilitates the transition of viral capsids across the nuclear envelope. This process of site-specific lamin phosphorylation is a very typical and common property of herpesviral NEC activities [4,7,8]. Beyond this, the phosphorylation of further NEC-relevant proteins is also an important hallmark of nuclear egress [9,10,11,12]. In our recent study, we demonstrated the oligomeric interaction of the HCMV core NEC proteins, a finding that directly referred to the hexameric NEC assemblies identified by our crystallization-based structural analyses [13,14,15]. The oligomeric interaction properties of pUL50–pUL53 were shown by in vitro assembly and coimmunoprecipitation approaches. Here, we applied a chemical protein cross-linking approach and demonstrated the oligomeric state of the HCMV core NEC using material from HCMV-infected or plasmid-transfected cells. Moreover, the association of cyclin-dependent kinases (CDKs) were detected on this basis, and the use of CDK inhibitors provided the first indication that these NEC-specific processes are sensitive to inhibitors of protein phosphorylation.

## 2. Materials and Methods

### 2.1. Cell Culture

Primary human foreskin fibroblasts (HFFs) were cultivated in minimal essential medium (MEM). Human embryonic kidney epithelial 293T cells (HEK 293T, CRL-3216, ATCC, Manassas, VA, USA) were cultivated in Dulbecco’s modified Eagle medium (DMEM, 11960044, Thermo Fisher Scientific, Waltham, MA, USA). Cell culture media were supplemented with 10% fetal bovine serum (FBS, F7524, Sigma Aldrich, St. Louis, MO, USA), 1× GlutaMAX^TM^ (35050038, Thermo Fisher Scientific) and 10 μg/mL gentamicin. All cells were incubated at 37 °C, 5% CO_2_ and 80% humidity.

### 2.2. Plasmid Constructs

Expression plasmids coding for HCMV pUL50 or pUL53, optionally carrying tags (HA, His or V5), were generated by PCR amplification of the UL50 or UL53 open reading frames (ORF), using the templates described earlier [16]. Oligonucleotide primers containing the tag sequences were purchased from Biomers (Ulm, Germany). The primers were amplified via PCR resulting in the ORFs becoming fused with C-terminal tags. Vent DNA polymerase (New England Biolabs, Ipswich, MA, USA) was used to perform PCR with 36 cycles (denaturation at 94 °C for 40 s, annealing at 58 °C for 40 s and polymerization at 72 °C for 90 s). PCR products were cleaved with the restriction enzymes *Eco*RI/*Xho*I and were inserted into the vector pcDNA3.1 (Invitrogen).

### 2.3. Protein Kinase Inhibitors

Cyclin-dependent kinase (CDK) inhibitors used in the present study were provided by several sources: R25/alsterpaullone from GPC Biotech AG (Martinsried, Germany); CDK2 Inh II from Abcam (Cambridge, UK); SEL120 from Selleck Chemicals GmbH (Munich, Germany). In addition, the viral kinase pUL97 inhibitor maribavir (MBV) was synthesized by Shanghai PI Chemicals Ltd. (Shanghai, China). All compounds were dissolved in dimethylsulfoxide (DMSO) according to manufacturers’ instructions and stored at −20 °C.

### 2.4. Antibodies

Monoclonal (mAb) and polyclonal (pAb) antibodies were used to detect the following proteins: mouse mAb-CDK1 (MA5-11472, Thermo Fisher), rabbit pAb-CDK2 (sc-163, Santa Cruz, Dallas, TX, USA), rabbit pAb-AMPKα (2532, Cell Signaling Technology, Danvers, MA, USA), rabbit mAb-lamin A/C (EPR4100, Abcam, Cambridge, UK), rabbit pAb-aldolase (Santa Cruz), mouse mAb-MCP (28-4), mouse mAb-HA (clone HA-7, Sigma-Aldrich), rabbit pAb-HA (T501, Signalway Antibody, College Park, MD, USA), mouse mAb-His (MA1-21315, Thermo Fisher), rat mAb-HA-HRP (3F10, Roche, Basel, Switzerland), mouse mAb-V5 (R960-25, Invitrogen, Waltham, MA, USA), mouse mAb-UL50.01 and mAb-UL53.01 (kindly provided by Tihana Lenac Rovis and Stipan Jonjic, Univ. Rijeka, Croatia), rabbit pAb-UL97 (kindly provided by Detlef Michel, Univ. Ulm, Germany). Alexa Fluor 555- and 647-conjugated antibodies were used as secondary antibodies for indirect immunofluorescence staining (Molecular Probes, Eugene, OR, USA) and horseradish peroxidase-conjugated anti-mouse and -rabbit (Dianova, Hamburg, Germany) for Western blot (Wb) analysis.

### 2.5. In vitro NEC Assembly Assay for Chemical Cross-Linking followed by Immunoprecipitation (XL-IP)

The 293T cells were cultivated in 10 cm petri dishes with a density of 5 × 10^6^ cells per dish overnight prior to transient transfection with the individual plasmids by polyethylenimine (PEI)-DNA complexes (Sigma Aldrich) as described previously [17]. Transfected cells were harvested two to three days post transfection (d p.t.) for in vitro assembly-based CoIP. Cell pellets were resuspended in 600 µL HEPES buffer (1 M, pH 7.5) including protease inhibitors such as 20 µM phenylmethylsulfonyl fluoride (PMSF). Lysis was achieved by sonication on ice with 80% duty cycle for at least 20 s and lysis was continued on ice for an additional 20 min. The suspensions were then centrifuged at 4 °C for 10 min at 14,000 rpm to remove insoluble debris, and thereafter 100 µL of the different homogeneous lysates were combined for protein assembly in 1.5 mL microcentrifuge tubes and HEPES buffer was used to equalize identical volumes. The assembly reactions were incubated at 4 °C overnight under rotation. On the next day, disuccinimidyl suberate (DSS; CAS-No. 68528-80-3, Thermo Fisher Scientific, Waltham, MA, USA), which was utilized as chemical cross-linker in solution, was added at various concentrations to stabilize protein assemblies by covalent conjugation, and further incubated at 37 °C for 1 h. The cross-linking reaction was quenched by adding CoIP buffer (50 mM Tris/HCl pH 8.0, 150 mM NaCl, 5 mM EDTA and 0.5% NP40) and incubated at 37 °C for at least 30 min. Thereafter, the cross-linked protein complexes were either directly used for analysis by SDS-PAGE/Wb (XL-lysates), or, optionally, were incubated with Dynabeads™ Protein A (10002D, Thermo Fisher Scientific) coupled with tag-specific antibodies to achieve cross-linked protein immunoprecipitation (XL-IP; antibody concentrations according to manufacturers’ instructions) at 4 °C for approximately 4 h. Finally, the beads were washed to remove the unbound protein fraction, before cross-linked samples were analyzed by Wb staining procedures to detect protein–protein interactions.

### 2.6. Intracellular Approach of Chemical Cross-Linking Using Material from Transient Transfection

The 293T with a density of 5 × 10^6^ cells were seeded in 10 cm petri dishes and incubated at 37 °C overnight before transfection. As described previously [13], PEI transfection was performed with single plasmids or combined plasmids to transiently express the differentially tagged pUL50 and pUL53. Two to three days after incubation at 37 °C, the transfected 293T cells were harvested and washed with HEPES buffer prior to an intracellular application of the cross-linking as described above. After a quenching step, cross-linked samples were used to detect protein–protein interactions by Wb analysis.

### 2.7. Intracellular Cross-Linking Using Material from Virus Infection

Viral stocks of HCMV strain AD169 and recombinant AD169 expressing an HA-tagged pUL50 (AD169 UL50-HA) were propagated on HFFs. The infectious titers were determined by standard plaque assay. HFFs were cultivated in 6-well plates with a cell density of 2.5 × 10^5^ at the day (d) before infection. After 90 min of viral adsorption, the inocula were removed, media were refreshed and cells were further cultivated at 37 °C for 5 d. Under conditions of inhibitor treatment, infected cells were treated with indicated concentrations of compounds, starting on 3 d post infection (d p.i.) after the first-round onset of viral replication. The infected cells were harvested 5 d p.i. and resuspended in HEPES buffer. Each cross-linked sample was derived from one cell lysate combining three wells of a 6-well plate. To initiate the cross-link reaction, various concentrations of DSS were added and incubated at room temperature (RT) for 30 min under rotation. Thereafter, CoIP buffer was added to the cross-linking samples to quench the reaction. The protein–protein interactions of total lysates were assessed by Wb. Optionally, cross-linked samples were subjected to IP and analyzed accordingly. Hereby, the cross-linked samples were subjected to sonication and homogeneous lysates were incubated with antibody-coated Dynabeads™ Protein A at 4 °C for 2 h under rotation. Thereafter, beads were washed and the interaction complexes were detected by SDS-PAGE/Wb staining procedures.

### 2.8. Virus-Specific Quantitative Polymerase Chain Reaction (qPCR)

HFFs were seeded on cover slips in 6-well plates at a density of 2.5 × 10^5^ one day prior to infection with recombinant green fluorescent protein (GFP)-expressing HCMV (AD169-GFP). After 90 min of viral adsorption, media with inhibitor compounds at indicated concentrations were refreshed, before continued incubation at 37 °C for 5 day. On day five, the infected cells were utilized for indirect immunofluorescence assay and viral genome equivalents of the supernatants of HCMV-infected HFFs were quantitated by qPCR. For this purpose, the supernatants were centrifuged at 1500x *g* and digested with proteinase K at 56 °C for 1 h to release viral genomes from particles. The reactions were stopped at 95 °C for 5 min. The amount of extracellular viral genomic loads was measured in 5 µL of each sample by real-time PCR (TaqMan-PCR). Two primers, namely 5′CMV (AAGCGGCCTCTGATAACCAAG) and 3′CMV (GAGCAGACTCTCAGAGGATCGG), which anneal to a sequence within the major immediate early gene region of HCMV, were employed to amplify the viral genomes. Furthermore, a FAM/TAMRA-labeled probe was utilized for quantitative measurement. The viral load of a sample treated with the solvent DMSO, instead of an inhibitor, served as a reference control. HCMV genome equivalents from compound-treated viral supernatants were calculated as % of the DMSO control.

### 2.9. Indirect Immunofluorescence Assay and Confocal Laser-Scanning Microscopy

HCMV (AD169-GFP)-infected cells were fixed with 10% formalin at RT for approximately 8 min. Thereafter, HFFs were permeabilized using 0.2% Triton X-100 in PBS for 15 min, prior to a blocking step using Cohn II immunoglobulin fraction. Permeabilized cells were then incubated with indicated primary antibodies at 37 °C for 60 min, followed by a double-staining with secondary antibodies conjugated with Alexa Fluor^®^ 555 and Alexa Fluor^®^ 647. DAPI Vectashield mounting medium was utilized to counterstain the cellular nuclei. Data for immunofluorescence were collected using a TCS SP5 confocal laser-scanning microscope (Leica Microsystems, Wetzlar, Germany). Images of a confocal plane were taken with a line average of 3 at magnification of 1024 × 1024.

### 2.10. Cytotoxicity Assay

HFF cells were seeded in a 96-well plate with a density of 1.35 × 10^4^ cells per well and incubated at 37 °C for 24 h. The cultured HFFs were treated with inhibitor compounds (R25/alsterpaullone, MBV, CDK2 inh II or SEL120), at concentrations ranging from 0.2 µM to 100 µM, and incubated at 37 °C for 7 d. A sample without compound (DMSO) served as a negative control and a sample with 1 µM of staurosporine (STP) used as a cyctotoxic positive control. The Neutral Red solution (40 µg/mL, Sigma Aldrich, N2889) was added to the cells on d 7 and incubated at 37 °C for 2–4 h. Finally, destain solution (ethanol/water/acetic acid, 50:49:1) was added, and the uptake of Neutral Red was quantitated by measuring the excitation/emission of fluorescence at 560/630 nm.

### 2.11. Nuclear Egress Assay

HFFs were cultivated at a confluency of approximately 90% in a T-75 cm^2^ flask and were infected with HCMV strain AD169. Infected cells were treated with indicated concentrations of MBV following 90 min of viral adsorption and were further incubated at 37 °C for 6 d. Cells were harvested and a preliminary fractionation was performed by the REAP method (Rapid, Efficient And Practical method [18]), resulting in whole cell lysates (W), cytoplasmic fractions (C) and nuclear fractions (N). Thereafter, fractions W and N were further lysed by 10 freeze-and-thaw cycles (liquid nitrogen and 37 °C water bath). A small amount (25 µL) of each fraction was kept for Wb detection prior to digest of the main fraction volumes by DNase (04716728001, Sigma Aldrich) at 25 °C for 30 min. Thereafter, all fractions were purified by the use of a 30% sucrose cushion via centrifugation at 20,800 rcf at RT for 3 h. The pellets were harvested and resuspended in 100 µL PBS as previously described [19].

## 3. Results

### 3.1. A Cross-Linking Approach Directed to the HCMV-Specific NEC Indicates Oligomerization of the Viral Core NEC Proteins and Multicomponent Assemblies with Host CDKs

**Figure 1:**
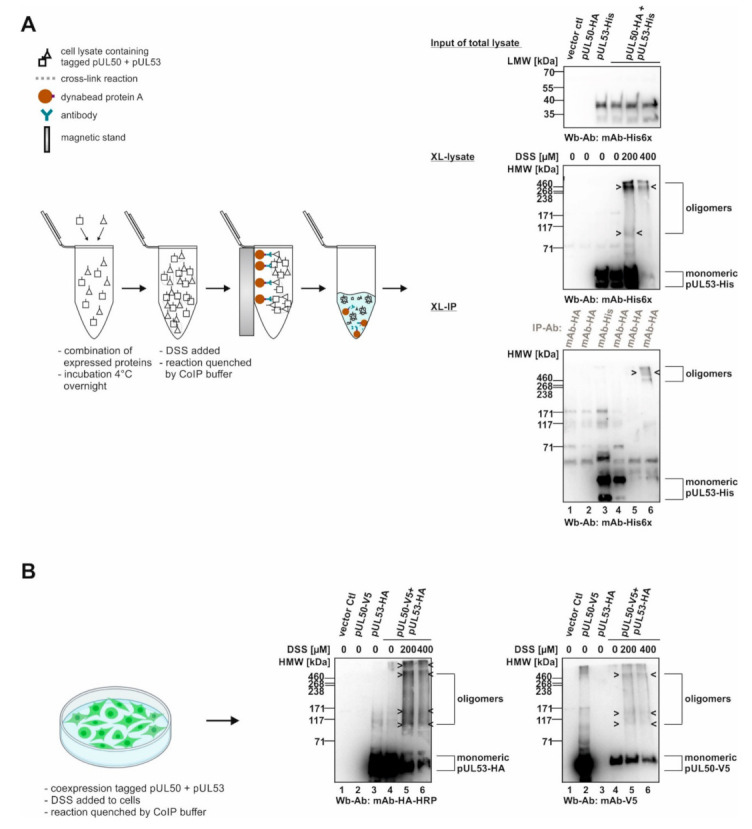
Schematic depiction of the cross-linking approach (XL) using transiently expressed proteins: signals of core NEC protein oligomerization. (**A**) In vitro NEC assembly assay for chemical cross-linking (XL-IP): the single transient expression of tagged pUL50 and pUL53 were assembled and DSS was utilized to stabilize protein–protein interactions by covalent conjugation. Oligomer formation was demonstrated by immunoprecipitation and Wb detection was achieved using tag-specific antibodies. (**B**) Intracellular approach of chemical cross-linking (XL): core NEC oligomers can be directly detected via cross-linking the coexpressed tagged pUL50 and pUL53 from total lysates of 293T cells using Wb analysis. HMW/LMW, high/low molecular weight marker.

Recently, we demonstrated the property of HCMV-specific core NEC proteins, pUL50 and pUL53, to form oligomeric assemblies. These had been detected using in vitro assembly approaches, coimmunoprecipitation settings with materials from plasmid-transfected cells, and X-ray studies on crystallized protein complexes [13,15]. In the present analysis, we addressed the question whether pUL50–pUL53 assemblies may be similarly detectable by chemical cross-linking using materials either from plasmid-transfected or from virus-infected cells. To this end, we established appropriate conditions of a mild and selective cross-linking approach that allowed for the visualization of oligomeric forms of the core NEC, and likewise associated proteins within the extended multicomponent NEC, through SDS-PAGE separation and Wb staining. In a first step, we applied the DSS-based cross-linking (XL) procedure to detect oligomeric assemblies when we transiently coexpressed tagged versions of pUL50 and pUL53, then produced total cellular lysates and subjected the material to the DSS reaction (Figure 1). At the concentrations of 200 and 400 µM of DSS, oligomeric forms were identified in cross-linked preparations of the total lysates (XL-lysate; Figure 1A). Positive signals were also obtained with samples of mAb-HA-specific immunoprecipitation at the concentration of 400 µM DSS (XL-IP; Figure 1A). It should be noted that the obtained signal patterns were not limited to individual bands of defined dimeric, trimeric and oligomeric forms, but appeared quite complex showing additional intermediate bands. This was most probably due to the additional formation of posttranslational modifications of both NEC proteins (i.e., in particular, pUL53 was sometimes represented by up to five bands on Wb including phosphorylated forms [5,8,13,20]) and additionally by varieties of protein degradation. Nevertheless, the detection of oligomeric assemblies was highly reproducible, also when applying a second approach of intracellular chemical cross-linking, by DSS treatment of these proteins directly in non-lysed cells after transient transfection (Figure 1B). Under these conditions, a similar pattern of oligomeric pUL50–pUL53 assemblies became detectable through cross-linking using 200 or 400 µM DSS, whereby the quantities of the monomeric forms decreased in indirect proportion to the appearance of oligomers (Figure 1B, see labelings at the right).

**Figure 2:**
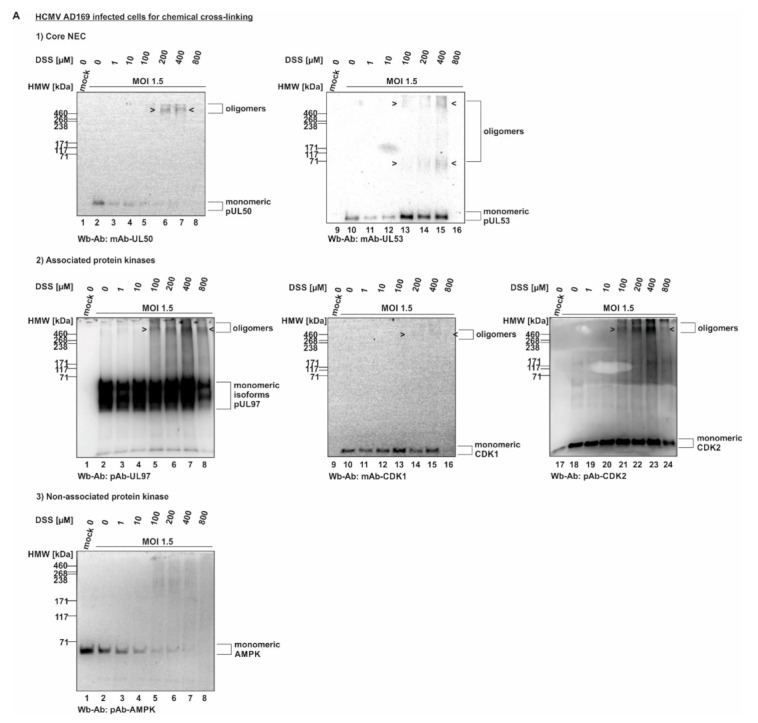

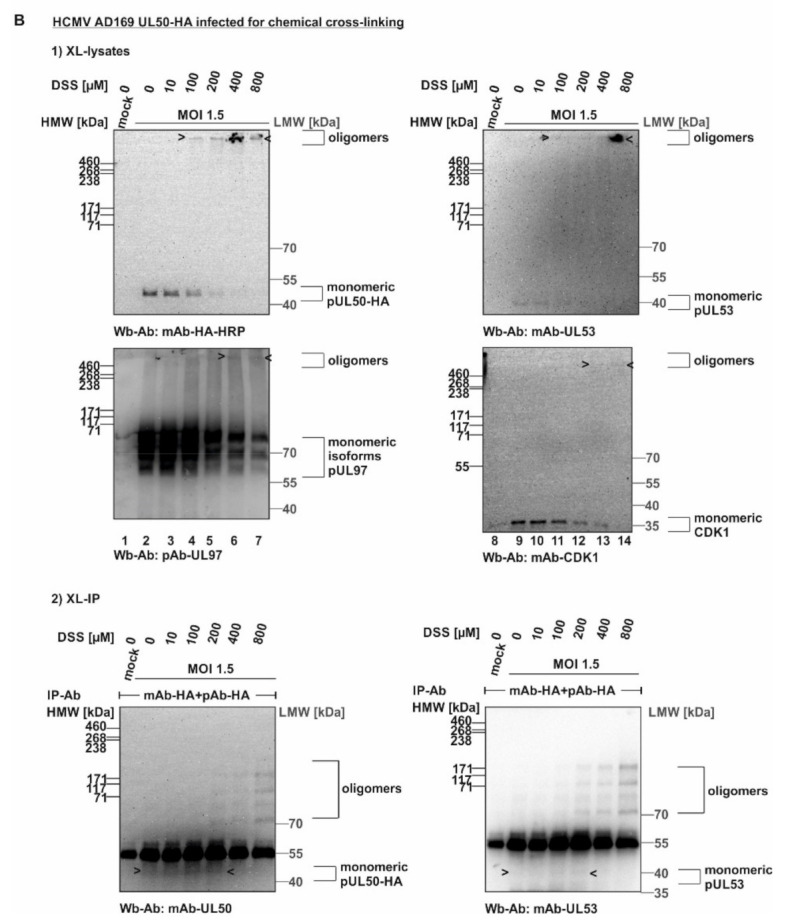
HCMV-infected cell proteins used to detect core NEC oligomerization and association of host cell kinases. (**A**) Human foreskin fibroblasts (HFFs) were cultivated in 6-well plates, used for HCMV AD169 infection at a multiplicity of infection (MOI) of 1.5 and harvested at 5 d p.i. Cross-linking was performed by addition of different concentrations of DSS. Core NEC oligomers and their associated protein kinases could be illustrated by Wb analysis. (**B**) HFFs were infected with HCMV AD169 UL50-HA at an MOI 1.5 and harvested at 5 d p.i. prior to performing cross-linking with DSS. Oligomeric formation and associated proteins were detected by Wb. Lower orders of core NEC oligomers were demonstrated by coimmunoprecipitation and Wb detection using the respective tag-specific antibodies. HMW/LMW, high/low molecular weight marker.

In a next step, the cross-linking procedure was extended to the use of proteins derived from HCMV-infected cells (Figure 2). For this purpose, primary human HFFs were used for infection with HCMV strain AD169, at a sufficiently high level of viral multiplicity (MOI 1.5), before total lysates were prepared and subjected to DSS treatment. Here again, oligomeric forms of both pUL50 and pUL53 were detectable (Figure 2A). Note that concentrations of 200 and 400 µM of DSS were found optimal for the detection of these signals, whereas lower (1, 10, 100 µM) or higher concentrations (800 µM) led to limited or negative signal quantities. In addition to the viral core NEC proteins, pUL50 and pUL53, we addressed the question, whether associated proteins of the multicomponent NEC might also be incorporated into oligomeric protein complexes in HCMV-infected cells. Here, the main focus of interest was directed to the viral and cellular protein kinases, pUL97, CDK1 and CDK2, representing known NEC-associated effector proteins [7,8,13,21]. An analysis of the DSS cross-linked samples on Wbs stained with pUL97-, CDK1- and CDK2-specific antibodies actually provided first evidence that these NEC-associated effectors may also be present in oligomeric assemblies (Figure 2A, middle panels/associated protein kinases). The quantity of these kinases was constantly low, i.e., signals remained at the detection limit, but was reproducible in experimental replicates. Interestingly, all three protein kinases indicated some partial association with higher-order protein complexes, although their direct containment within pUL50–pUL53-specific NEC oligomers could not be proven at this stage of investigation. In a specificity control setting, the cellular kinase AMPK (5′ adenosine monophosphate-activated protein kinase), which exerts a virus-supportive function independent from nuclear egress [22,23], was analyzed in parallel and did not show oligomers (Figure 2A, lower panel/non-associated protein kinase). Thus, this specificity control with the non-NEC-associated cellular kinase AMPK strongly argued against a nonspecific cross-linking of irrelevant proteins under these mild conditions of DSS treatment. Additional control settings with CDK9 (as another non-NEC-associated kinase as negative control) and lamin A/C (as an NEC-associated positive control) further supported the reliability of the cross-linking procedure (data not shown; for lamin A/C association, see references [4,5,7,21]).

In order to corroborate this finding, analogous experiments were performed by the use of a recombinant HCMV expressing the tagged version of pUL50-HA that had been characterized earlier [24]. By the use of HCMV AD169 UL50-HA (MOI 1.5), the DSS-specific oligomeric forms of pUL50, pUL53, pUL97 and CDK1 were confirmed (Figure 2B, upper panels), whereby the signal strength was limited in all cases. In order to intensify signal levels, we undertook an approach of additional immunoprecipitation of the cross-linked protein varieties (XL-IP). In fact, we obtained pUL50- and pUL53-specific signals of oligomers that increased along the applied DSS concentrations (Figure 2B, lower panels). However, the success of XL-IP was mostly restricted to the lower-order oligomeric forms of pUL50 and pUL53, so that no direct verification of the higher-order oligomeric forms obtained with total lysates (upper panels) was achieved. Nevertheless, these experiments show that pUL50 and pUL53 are basically capable of forming higher-order complexes, and thus, we further addressed the question whether these also include NEC-associated proteins, in particular protein kinases, putatively bound to NEC oligomers in HCMV-infected cells. So far, our data could not rule out that the CDK-specific signals in the cross-link samples may refer to other NEC-independent higher-order complexes with CDKs. To illustrate this point, we performed another in vitro assembly assay under the described conditions. In this setting, pUL53-Flag and pUL50-HA were separately expressed in transiently transfected 293T cells, before total lysates were prepared and combinations of these were incubated for core NEC in vitro assembly in the presence of endogenous CDK1 (Figure S1, input control of total lysates). These assemblies were subjected to cross-linking by DSS (200–400 µM) and used for HA-specific immunoprecipitation. Samples were taken before (Figure S1, XL-lysates) and after immunoprecipitation (XL-IP) and analyzed by SDS-PAGE/Wb using a CDK1-specific antibody. Oligomeric forms were detected in both cases, mostly visible under treatment with 400 µM DSS (Figure S1, XL-lysates and XL-IP, lane 4). Of note, the optimal concentration of DSS was different between the conditions chosen for individual experiments, e.g., Figure 1 and Figure 2. Most probably, this was based on the difference between overexpressed proteins by plasmid transfection (Figure 1) and lower protein levels obtained by HCMV infection (Figure 2B).

### 3.2. Importance of Cyclin-Dependent Kinases for the Efficiency of HCMV Replication

**Figure 3:**
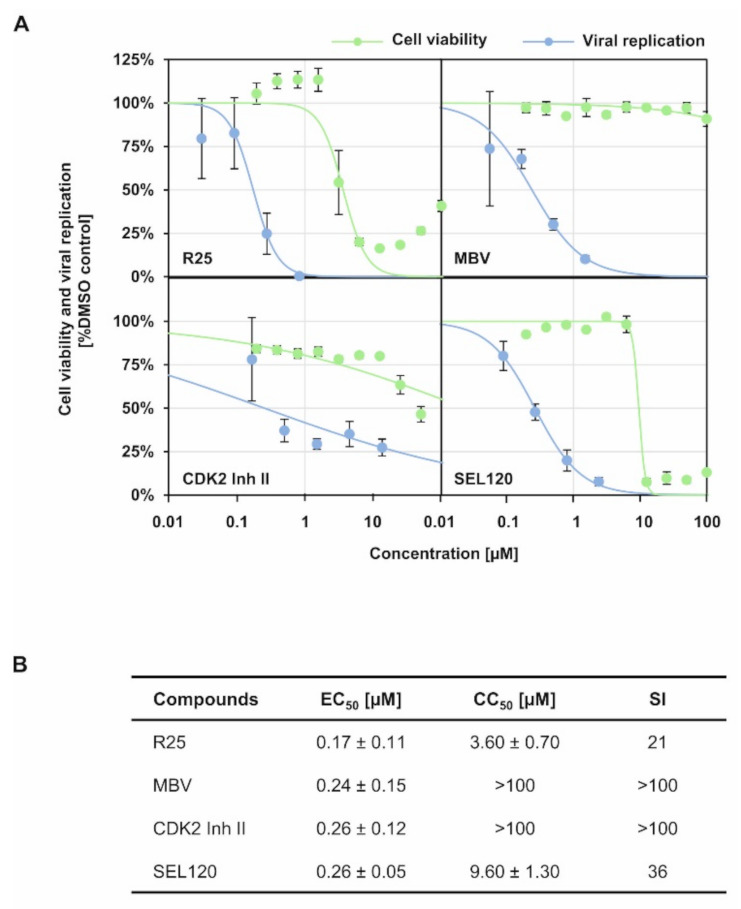
Anti-HCMV activity of CDK/vCDK inhibitors with known relevance for viral NEC regulation. (**A**) Virus yield assay was performed as described in Section 2.8 and anti-HCMV activity of indicated CDK/vCDK inhibitors was assessed (blue curve). In addition, cytotoxicity of the CDK/vCDK inhibitors on HFFs was determined as described in Section 2.10 and CC_50_ values were calculated (green curve). (**B**) Determination of values of EC_50_, CC_50_ and SI, as based on the data provided by panel (**A**). Mean values ± SD of triplicates are shown.

As far as the importance of regulatory host kinases, especially CDKs, for HCMV replication is concerned, our previous studies strongly emphasized the antiviral potency of pharmacological kinase inhibitors. Inhibitors of both kinase types, i.e., host CDKs, mostly CDK1/2/7/9, as well as the viral CDK ortholog pUL97 (vCDK), exert a strong antiviral effect against HCMV independent of viral strain and host cell type [8,25,26,27,28,29,30,31,32]. Moreover, a pronounced level of synergistic interaction has been proven for anti-HCMV drug combination treatments directed to vCDK/pUL97 and CDK inhibitors [33,34]. This finding is further illustrated by the fact that CDK1 and vCDK/pUL97 are able to phosphorylate both HCMV core NEC proteins pUL50 and pUL53 [8,35,36]. In this context, an obvious focus of the present study was the question whether the antiviral activity of CDK inhibitors may correlate with their putative interference with intranuclear NEC localization and oligomerization. To this end, we determined the anti-HCMV activity of inhibitors of human CDKs or vCDK/pUL97 with known relevance for viral NEC regulation (Figure 3), i.e., R25/alsterpaullone (CDK1/2/5), maribavir/MBV/Livtencity^TM^ (vCDK), CDK2 Inh II (CDK2) and, as a non-NEC-relevant control, SEL120 (CDK8). For all four drugs, a pronounced concentration-dependent profile of antiviral activity was detectable (Figure 3A), which was clearly distinguishable from the range of cytotoxic effects. For R25, MBV, CDK2 Inh II and SEL120, the mean values of EC_50_ were in the submicromolar range, with 0.17 ± 0.11 µM, 0.24 ± 0.15 µM, 0.26 ± 0.12 µM and 0.26 ± 0.05 µM, resulting in selectivity indices (SI) of 21, >100, >100 and 36, respectively (Figure 3B).

**Figure 4:**
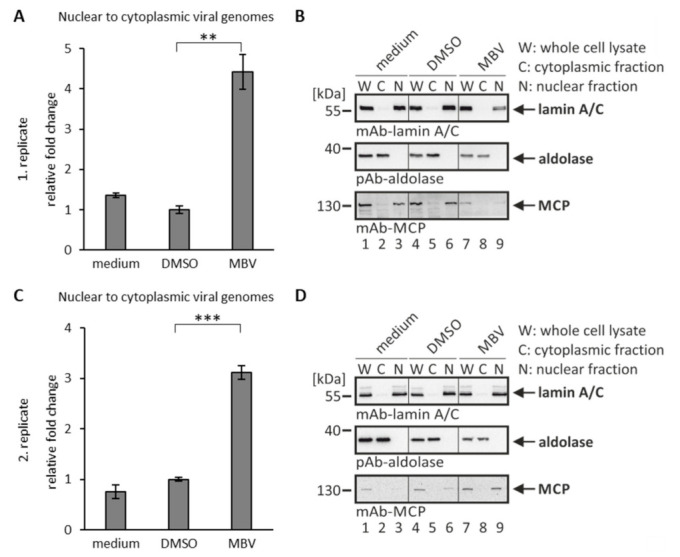
Nuclear egress assay based on cell fractionation and virus-specific qPCR: inhibition of nuclear egress by the viral kinase inhibitor maribavir. HFF cells were infected with HCMV AD169 at an MOI of 0.01 and treated with 5 µM MBV or DMSO as a solvent control. At 6 d p.i., cells were harvested and the nuclear egress assay was performed. (**A**,**C**) The qPCR measurements were performed in technical triplicates, so that viral genome copies of C and N fractions of the two depicted biological replicates were summed up and set to 100%. The fold change between the percentages of nuclear to cytoplasmic viral genomes was calculated. Mean values ± SD are given and the significance between DMSO and MBV was calculated by Student’s *t*-test (** *p* < 0.01; *** *p* < 0.001). (**B**,**D**) Samples taken after fractionation were subjected to Wb analysis using the indicated antibodies. W, whole cell lysate; C, cytoplasmic fraction; N, nuclear fraction.

As a next step of analysis, we investigated the activity of antiviral drugs using our recently established quantitative nuclear egress assay [19]. This assay is based on cell fractionation and isolation of viral genomic DNA from the cytoplasm of infected cells for quantitative assessment by qPCR. By the use of this nuclear egress assay, the efficiency of nucleocytoplasmic release of encapsided genomes of mutant HCMVs was investigated [9,19,37]. Here, we analyzed the putative effect of MBV in this assay. Based on the situation that viral pUL97, representing the target of MBV with proven selectivity [33], exerts major NEC-regulatory kinase activity [7,21,38], we assessed its inhibitory potency using the nuclear egress assay (Figure 4). In fact, the analysis demonstrated a quantitative nuclear egress-inhibitory activity at a concentration of 5 µM in HCMV-infected primary fibroblasts (Figure 4A). The reliability of assay conditions, in particular the success of cell fractionation, was visualized by Wb analysis using lysate and fraction samples in parallel (Figure 4B,D). Statistical significance of the egress-inhibitory effect of MBV compared to the solvent control (DMSO) could be demonstrated in two independent experimental replicates (Figure 4A,C; qPCR was performed in technical triplicates for each experiment). Based on the two depicted biological replicates (Figure 4A,C), the mean of each depicted experiment was then calculated to provide the relative changes in nuclear-to-cytoplasmic release of genome-encapsidated viral particles of 4.4 ± 0.4-fold (Figure 4A) and 3.1 ± 0.1-fold (C) for MBV versus DMSO, as well as 3.3 ± 0.3-fold (A) and 4.2 ± 0.2-fold (C) for MBV versus medium, respectively. Morever, another experiment was conducted with an MOI of 0.03, resulting in a very similar result (data not shown), thus supporting the MBV-specific block of HCMV nuclear egress at low MOIs of 0.01 and 0.03. This result further underlines the crucial role of the pUL97 kinase for viral nuclear egress and shows for the first time a clearly measurable inhibitory effect of MBV in this qPCR-based egress assay.

**Figure 5:**
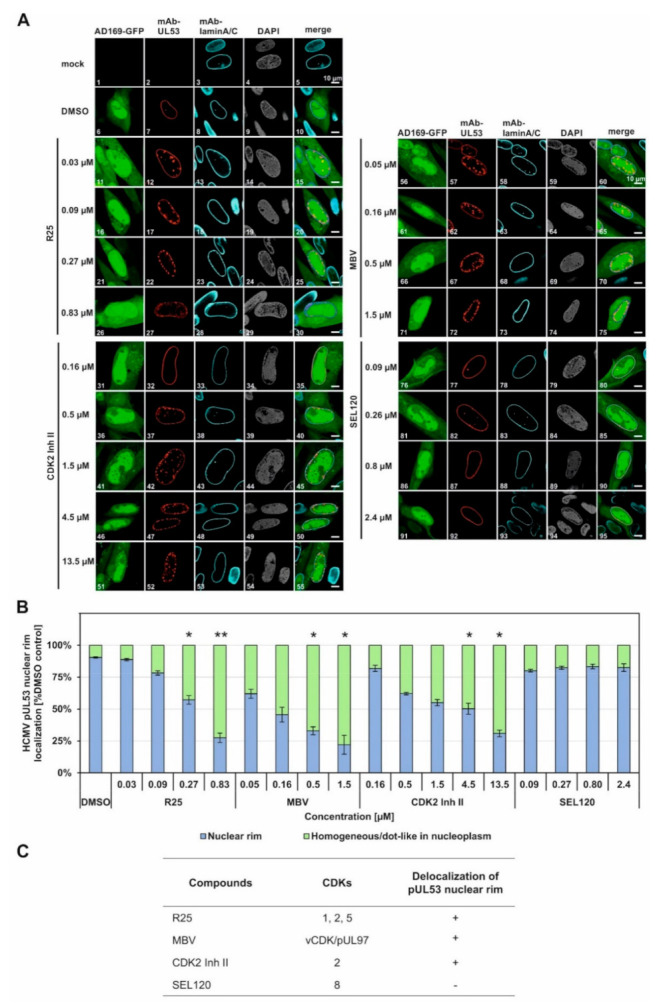
Effect of CDK/vCDK inhibitors on pUL53 nuclear rim localization. (**A**) Confocal imaging of NEC nuclear rim formation in HCMV AD169-GFP-infected fibroblasts under CDK/vCDK inhibitors treatment. Infected cells were fixed at 5 d p.i. and indirect immunofluorescence staining was performed for viral pUL53 and cellular lamin A/C. Counterstaining of the autofluorescent AD169 and the nuclei (DAPI) are indicated and a merge of HCMV AD169-GFP, pUL53 and lamin A/C is given on the right. Note the concentration-dependent inhibition of normal pUL53 nuclear rim localization by R25, MBV and CDK2 Inh II but not by the control compound SEL120. (**B**) Quantitation of the inhibitor-mediated decrease of pUL53 nuclear rim localization was performed by visual microscopic counting. The criteria of counting were based on areas of pUL53-positive cells that either comprised a perfect nuclear rim localization of pUL53 (positive cells, i.e., identical with DMSO control) or a loss of perfect nuclear rim localization by the occurrence of intranuclear speckles (negative cells). Several areas of positive cells (200 cells in mean) were used for the evaluations and mean values ± SD of counting in duplicate are shown. The significance between DMSO and CDK/vCDk inhibitors was calculated by Student’s *t*-test (* *p* ≤ 0.05; ** *p* ≤ 0.01). (**C**) Summary of the relevant effects of CDK/vCDK inhibitors on pUL53 localization.

It should also be mentioned, however, that an effect similar to MBV was not reproducibly obtained for other known inhibitors of viral nuclear egress, such as the recently identified core NEC-interfering small molecule merbromin (MBM; [13,39]). For MBM, the data obtained with the qPCR-based nuclear egress assay did not show unequivocal and robust indication for NEC-inhibitory activity (data not shown). A reason for this may be given by some limitation of separation precision residing within the procedure of cell fractionation. Nevertheless, another measurement of nuclear egress assay was performed with the antiviral CDK inhibitors described in the present study (Figure S2). Notably, for those CDK inhibitors that showed an anti-HCMV activity with known relevance for viral NEC regulation (Figure 3, Figure 4 and Figure 5), namely R25 and CDK2 Inh II, a reduced nuclear-to-cytoplasmic release of genome-encapsidated viral particles was measured by qPCR (Figure S2A,B; the reliability of cell fractionation was monitored by Wb analysis as shown in panel C). The relative fold changes induced by R25 or CDK2 Inh II treatment were less pronounced than those by MBV treatment, but effects were compatible with our data on R25-/CDK2 Inh II-mediated inhibition of NEC nuclear rim formation (see Section 3.3). To this end, a further NEC-specific analysis of CDK inhibitors was performed on a single-cell level by applying the confocal imaging-based nuclear rim formation assay [8,13]. Initial results of the inhibitory impact of R25 on viral core NEC assembly were provided before [8], but other kinase inhibitors behaved negatively so far. Here, we performed a closer confocal microscopic inspection and quantitative evaluation of the present CDK inhibitors of interest using this assay.

### 3.3. A Confocal Imaging-Based Assessment of Viral Nuclear Rim Signals Demonstrates the Inhibitory Potential of CDK Inhibitors on the Core NEC Formation in HCMV-Infected Cells

HCMV-infected HFFs were used for an indirect immunofluorescence staining of NEC-specific nuclear rim formation under CDK inhibitor treatment (Figure 5). The rim signal pattern that represents the regular structure of the nuclear envelope was visualized by control staining with a lamin A/C-specific antibody, the regular nucleoplasmic space by DAPI counterstaining, and virus-positive cells (infectecd by HCMV strain AD169-GFP) by nucleocytoplasmic autofluorescence of the viral green fluorescent protein (GFP). As the central marker of this assay, viral core NEC protein pUL53 was immunostained by the use of mAb-UL53 (Figure 5A, see top-line labels). Under normal conditions, i.e., under solvent control treatment in the absence of inhibitor (DMSO), the pUL53 signal showed a marked nuclear rim signal that marked the entire ring-like structure of the nuclear envelope in the confocal representation. Under conditions of a drug-disturbed rim localization, the pUL53 signal showed signal patterns of delocalization into the nucleoplasm, either in a homogeneous or dot-like in nucleoplasmic distribution. This effect of delocalization was noted for three of the four kinase inhibitors analyzed, namely, R25, CDK2 Inh II and MBV, while SEL120 did not exert a NEC-directed delocalization activity (Figure 5A, see labels at the left). This inhibitor effect was observed in a concentration-dependent manner that indicated an increase of the cell numbers, comprising a pUL53 nuclear rim delocalization, along with the rising drug concentrations applied (Figure 5A, images 12, 17, 22, 27 for R25 of 0.03 µM to 0.83 µM; images 32, 37, 42, 47, 52 for CDK2 Inh II of 0.16 µM to 13.5 µM; images 57, 62, 67, 72 for MBV of 0.05 µM to 1.5 µM). Interestingly, the CDK8 inhibitor SEL120, which exhibited a similar efficacy of antiviral activity as the other CDK inhibitors (compare Figure 3B), did not induce an alteration of pUL53 localization; thus, it appears to act in a different, NEC-independent mechanistic manner (images 77, 82, 87, 92). As the central finding, a quantitative evaluation of the cell numbers that comprised an inhibitor-mediated effect onto pUL53 confirmed the substantial delocalization activity in the case of R25, MBV and CDK2 Inh II, but not SEL120 (Figure 5B). The range of statistical significance of these effects was determined, as indicated by asterisks. This finding strongly pointed to those types of CDK, for which a relevance of NEC regulation had already been postulated, i.e., CDK2, CDK1 and the CDK2-related vCDK/pUL97 (Figure 5C), while other types such as CDK8 might have minor or no NEC-specific importance. We also investigated the colocalization of NEC-associated proteins such as pUL97, CDK1 and CDK2. It should be emphasized, however, that the demonstration of this protein kinase association with the HCMV core NEC is a challenging task, which has also been addressed before by using confocal imaging approaches, but without the result of obtaining a marked rim staining. In this context, our relevant data for viral pUL97 and cellular CDK1 were published earlier [13,40,41]. Interestingly, even the highly sensitive method of tandem-MS-based proteomics using NEC coimmunoprecipitates could not detect CDK association, but was restricted to pUL97 as the only detectable kinase signal [5]. Thus, the XL-approaches in the present study provide a first technical option to visualize these CDK associations that were previously postulated by functional assays [4,8,13,23].

### 3.4. CDK Inhibitors Show Effects towards the CDK Association with Oligomeric HCMV Core NEC

**Figure 6:**
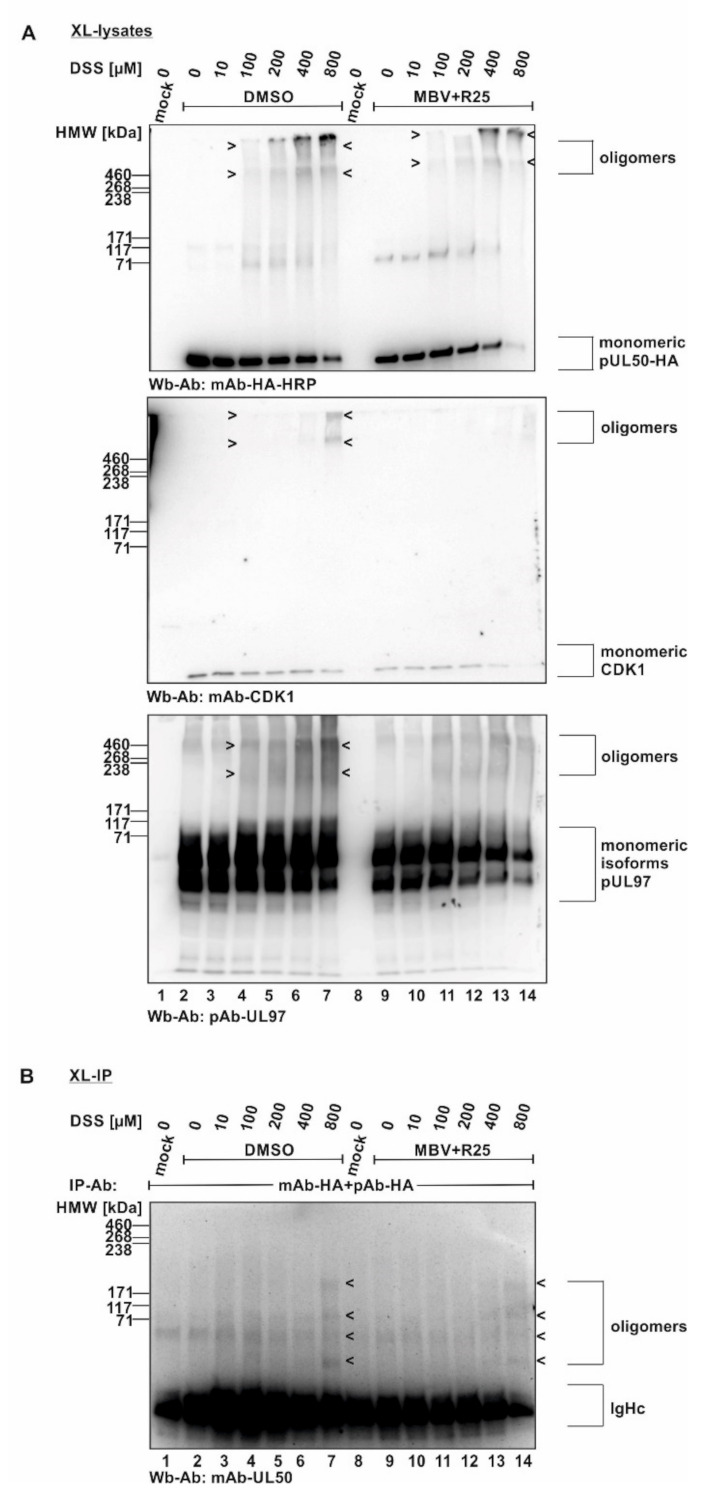
Inhibitory effect of kinase inhibitors on the association of CDK1 with oligomeric HCMV core NEC. (**A**) Proteins derived from total cellular lysates. (**B**) Proteins derived from coimmunoprecipitates. HFFs were seeded in 6-well plates, used for HCMV AD169 UL50-HA infection at an MOI of 1.5 and treated with the mixture of R25 and MBV at 3 d p.i. Cells were harvested at 5 d p.i. and cross-link was performed by addition of different concentrations of DSS. Core NEC oligomers and their associated protein kinases could be illustrated by Wb analysis. Different order of core NEC oligomers could be demonstrated by coimmunoprecipitation and Wb detection using the respective tag-specific antibodies.

Based on the identified NEC delocalization effect of CDK inhibitors, we next addressed the question whether these CDK inhibitors might exert an effect on the oligomeric assembly of the core NEC or on the association between the NEC and CDKs. Due to the finding that MBV and R25 showed most pronounced effects on the core NEC pUL53 nuclear rim localization, these two drugs were further investigated using the XL-lysates/XL-IP assays. For this purpose, a similar experimental setting was performed as shown in Figure 2. HFFs were used for infection with HCMV AD169 UL50-HA, harvested at the time point indicated, before total lysates (XL-lysates) were subjected to DSS-conferred cross-linking (Figure 6). The specific point in this experiment was the addition of kinase inhibitors MBV + R25 prior to the cross-linking reaction, i.e., a combined inhibitory activity against CDKs 1, 2, 5 and vCDK/pUL97 (Figure 6A, lanes 8–14). As compared to the DMSO control (lanes 1–7), no marked impact of the inhibitors was exerted to core NEC oligomerization (see upper panel, oligomers of pUL50-HA). Notably, however, the postulated association of CDK1 with these oligomeric forms was substantially reduced under MBV + R25 treatment (Figure 6A, middle panel, lanes 12–14) and the viral kinase pUL97 showed some partial reduction of oligomeric signals (lower panel). This finding indicated that the activity of kinase inhibitors may produce a negative effect on kinase association with the NEC. Although we cannot fully exclude the possibility that both NEC-associated and NEC-independent higher-order complexes with CDKs may additionally be detected under these conditions, the comigration with NEC bands under these DSS concentrations, together with findings in Figure S1, Figure 2, Figure 4 and Figure 6, argue for this interpretation. The oligomeric assembly of viral core NEC proteins, on the other hand, seemed not to be influenced by inhibitor treatment, and this conclusion was also confirmed by an additional immunoprecipitation of the cross-linked samples (XL-IP). Here, the cross-link-specific bands observed for pUL50 occurring under 800 µM of DSS were not found to be reduced under MBV + R25 inhibitor treatment (Figure 6B, lanes 7 and 14).

**Figure 7:**
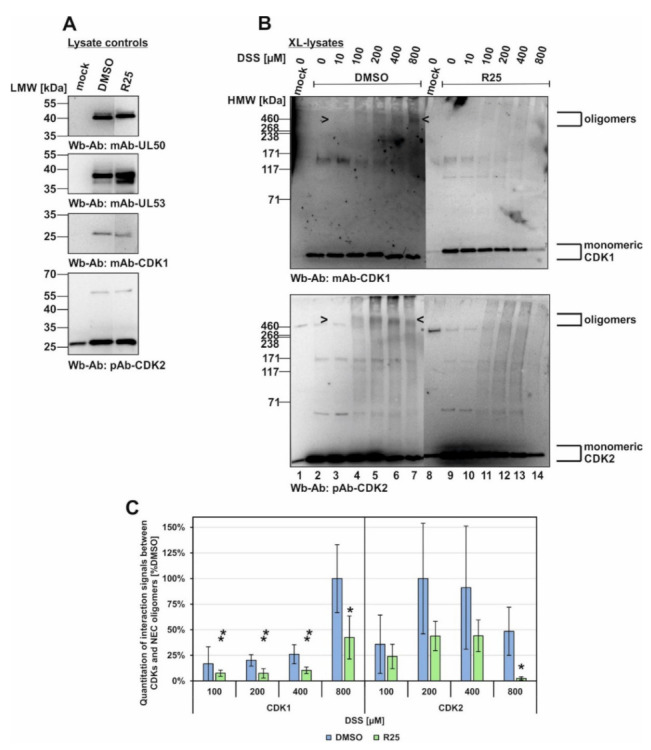
Inhibitory effect of kinase inhibitors on the association of CDK1 and CDK2 with oligomeric HCMV core NEC. HFFs were seeded in 6-well plates, used for HCMV AD169 UL50-HA infection at an MOI of 1.5 and treated with R25 at 3 d p.i. Cells were harvested at 5 d p.i. and cross-link was performed by addition of different concentrations of DSS. (**A**) Lysate controls were kept before DSS addition and expression levels of the individual proteins were monitored by Wb. (**B**) The associated protein kinases of cross-linked lysates were additionally detected by Wb analysis (see oligomeric forms marked by arrow-heads). (**C**) Quantitation of the band intensities was performed by densitometry (AIDA Image Analyzer v.4.23 software). All determinations were made in quadruplicate by using two identical Wbs (one of each shown under (**B**)). The mean values of CDK band intensity were corrected against the highest band intensity under DMSO control treatment. Statistical significance was calculated by Student’s *t*-test (* *p* ≤ 0.05; ** *p* ≤ 0.01).

Next, the inhibitor treatment was narrowed down to R25 only, due to the fact that so far the main blocking effect had been observed for CDK1 (Figure 7). In this experiment, the conditions were basically kept unaltered compared to Figure 6 (XL-lysates, DSS cross-linking upon R25 treatment), and the parallel Wbs with lysate controls showed comparable levels of the relevant proteins (Figure 7A). These data show that the inhibitor prevented oligomerization of CDK1 and CDK2 (Figure 7B,C). Concerning the postulated association of CDKs with the viral core NEC oligomers, however, no clear evidence could be provided at this stage of investigations. A quantitation of the oligomer signal reduction was then performed by densitometry using two identical Wb replicates of these samples (mean values ± SD of densitometry in quadruplicate; Figure 7C). Maximum signal value of each CDK1 or CDK2 panel was taken as 100% and the respective drug-mediated reduction was indicated. Thus, a pronounced reduction of both CDK1 as well as CDK2 signals was measured for R25 treatment. This result strongly supports our earlier notion that inhibition of kinase activity alters the protein interaction properties of CDKs. Although not proven by this approach, the results were compatible with an influence of kinase activity on the multicomponent NEC composition.

**Figure 8:**
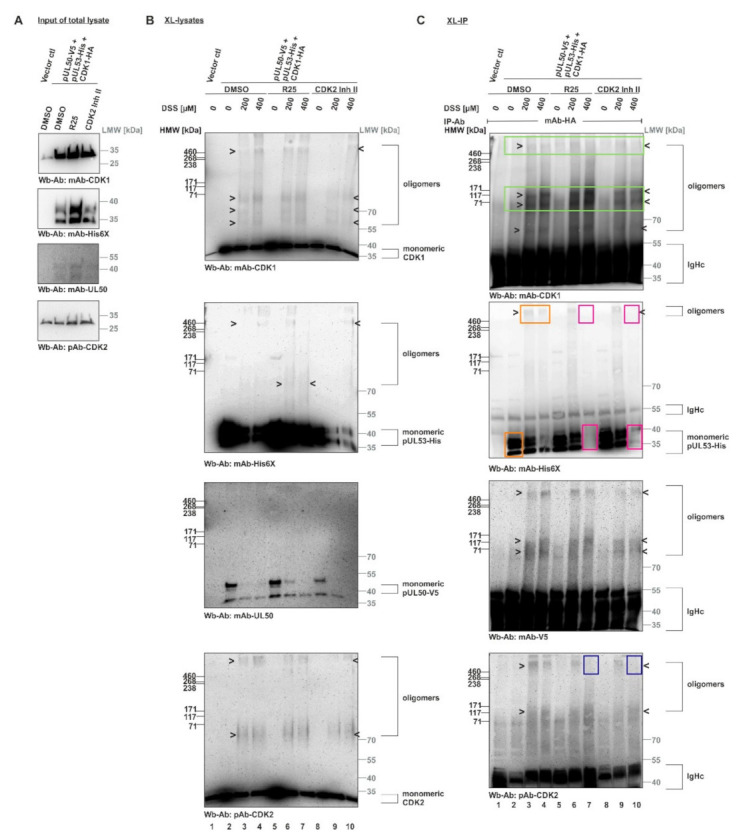
In vitro assembly XL-IP assay demonstrating the HCMV core NEC–CDK interaction and its sensitivity to CDK inhibitors. The 293T cells were cultivated in 10 cm petri dishes and used for transient transfection. Cells were harvested at 2 d p.t. In vitro assembly assay was performed by addition of 3 µM of the CDK inhibitors R25 or CDK2 Inh II to lysates during the assembly process of pUL50-V5, pUL53-His and CDK1-HA. DMSO treatment was used as a reference control. (**A**) Input samples of total lysates were collected before DSS addition in order to monitor the expression levels of the individual proteins by Wb. (**B**) Core NEC and associated protein kinases of cross-linked lysates were specifically detected by Wb using the indicated antibodies. (**C**) The interactions of core NEC–CDK were demonstrated by coimmunoprecipitation and Wb detection using the respective tag-specific antibodies (green frames, precipitation control of CDK1; orange frames, pUL53–CDK1 interaction; red frames, inhibition of pUL53–CDK1 interaction under R25 or CDK2 Inh II treatment; blue frames, R25-/CDK2 Inh II-mediated reduction of CDK2 association). HMW/LMW, high/low molecular weight marker.

Finally, we performed an in vitro assembly assay with XL-IP to further address the question of core NEC–CDK interaction by the use of overexpressed CDK1-HA (Figure 8). For this purpose, tagged proteins were produced by transient expression in single-transfected 293T cells, before they were combined into in vitro assembly settings (pUL50-V5 + pUL53-His + CDK1-HA). Samples of these combined total lysates were treated with the CDK inhibitors R25 or CDK2 Inh II, as indicated, before input controls were collected and subjected to a control SDS-PAGE/Wb detection (Figure 8A). Then, the reaction settings were subjected to DSS cross-linking in order to demonstrate the formation of oligomers, in addition to the monomeric forms, as demonstrated by Wb stainings performed on the XL-lysates (Figure 8B). Ultimately, CDK1-HA was immunoprecipitated by the use of mAb-HA and the coimmunoprecipitation of proteins was analyzed by the use of these XL-IP samples (Figure 8C). The success of CDK1-specific XL-IP was verified by the detection of its oligomeric forms (Figure 8C, upper panel, marked by green frames; note that the monomeric CDK1 signals were blacked out by IP-produced IgHc immunoglobulin heavy chains). Importantly, a strong and specific coimmunoprecipitation signal (absent from the vector control, lane 1) was obtained for the monomeric pUL53-His (0 µM DSS; lane 2, second upper panel, orange frame) and the oligomers (200–400 µM DSS; lanes 3 and 4, orange frames). Likewise, a cross-link signal was obtained for oligomeric bands of pUL50-V5 (second lower panel; the monomeric form was blacked out by IgHc). This finding indicated a direct interaction between the viral core NEC proteins and host CDK1. As another important finding, the treatment with R25 (lane 7) or CDK2 Inh II (lane 10) induced a loss of the oligomeric and monomeric pUL53 XL-IP signals under conditions of 400 µM DSS (second upper panel, red frames). This strongly suggested a sensitivity of the pUL53–CDK1 interaction towards CDK activity. Notably, the signals of pUL50 interaction with CDK1 showed a comparatively low sensitivity to these inhibitors, suggesting pUL50 association with CDK1 may be less dependent on kinase activity than pUL53 association (second lower panel). As an additional aspect, a cross-link-mediated association of the oligomeric forms of CDK2 was also detectable in the CDK1-specific XL-IP samples (lowest panel) and, similar to pUL53, a reduction of interaction signals was reproduced by R25 or CDK2 Inh II treatment (lanes 7 and 10, blue frames). Combined, these data support our postulate that the oligomerization of HCMV core NEC includes the association with host CDK1 and CDK2 and that these association processes are sensitive to kinase inhibitors.

## 4. Discussion

With the present study we provide further evidence for the pronounced oligomerization property of the HCMV-specific core NEC proteins, pUL50 and pUL53, through an approach of chemical protein cross-linking. In particular, the study thereby underlines that the oligomeric assemblies of the core NEC proteins are associated with host kinases and show sensitivity to selected kinase inhibitors, exerting a strong antiviral activity. The experimental findings lead to the following conclusions: (i) the oligomeric state of the HCMV core NEC can be demonstrated using material from both HCMV-infected or plasmid-transfected cells by applying chemical cross-linking, (ii) the NEC association of host kinases, specifically CDKs 1 and 2, was shown with the cross-linked multicomponent NECs, and (iii) specific small molecules directed to these NEC-associated kinases exerted NEC-inhibitory properties and antiviral activity.

As far as the oligomerization of the pUL50–pUL53 complex, or related herpesviral core NECs, is concerned, three different lines of evidence pointed to this property. First, the electron microscopic detection of intranuclear structures of HSV-1-specific NEC arrangements strongly suggested oligomerization. In particular, the ultrastructural architecture of alphaherpesviral NEC in the form of hexagonal arrays was postulated using a cell-based system that also demonstrated the formation of NEC-induced perinuclear vesicles [42]. Secondly, the 3D structures resolved from crystallized recombinant core NEC preparations revealed hexameric rings and their association to honeycomb-like lattices [15]. Models of the putative functional role of these higher-order NEC arrangements have been discussed in a number of articles [4,43,44]. Thirdly and recently, our studies identified the oligomeric pUL50–pUL53 properties by performing in vitro assembly approaches and, moreover, the antiviral activity of a novel NEC-directed small molecule, MBM, leading to the validation of this drug-accessible complex as an antiviral target.

**Figure 9:**
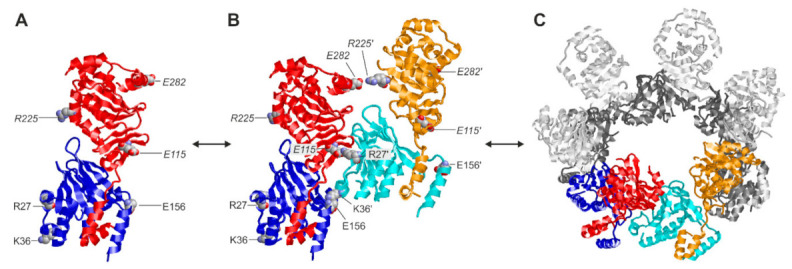
Structures of the different oligomerization states of the HCMV core NEC pUL50–pUL53. (**A**) Heterodimer of pUL50 (blue) and pUL53 (red). Charged residues that are involved in the formation of higher oligomers are shown in a space-filled presentation and labeled accordingly (labels in italics denote residues of pUL53). (**B**) Quaternary assembly of two heterodimers (blue/red and cyan/orange), which is stabilized by interaction between the negatively charged residues of the first heterodimer and the positively charged residues of the second heterodimer, resulting in two strong (*E115–R27*′, *E156–K36*′) and one weak (*E282*–*R225′*) interactions (labels with a prime denote residues of the second heterodimer). (**C**) Hexameric ring formed by six heterodimers. The colored heterodimers correspond to the assembly shown in (**B**).

Here, our novel data specifically illustrate this pronounced oligomerization property of the HCMV-specific core NEC on the basis of the chemical cross-linking approach. This finding raised the question about the stability of small oligomers formed by the interaction between individual pUL50–pUL53 heterodimers (Figure 9). This issue has been previously addressed by molecular dynamics simulations [45], comparing the stability of the interactions formed in a heterodimer (Figure 9A), a quaternary assembly of two heterodimers (termed dimer–dimer; Figure 9B) and a hexameric ring of heterodimers (Figure 9C). Based on our previously published data, the strength of the electrostatic interactions formed in the dimer–dimer and the hexameric ring proved to be very similar [45]. This indicates that the dimer–dimer, and in a similar manner probably other small oligomers as well, already exhibit a significant degree of stability that might be sufficient to be captured by the cross-linking. However, it cannot be ruled out that the oligomers observed in the present study result from a partial cross-linking within larger oligomers followed by dissociation of non-covalently linked subunits. These details need to be further elucidated by future studies.

As far as the functional role of pUL50–pUL53 oligomeric assembly is concerned, no clear data-based concept is available so far. Principally, it is an accepted model that the formation of hexameric assemblies and higher-order structural lattices provide a scaffold for the docking of nuclear capsids on their way to nucleocytoplasmic release [4,22,43,44]. Furthermore, the recruitment of a variety of viral and cellular NEC-associated effector proteins has been assigned to the multifaceted binding interfaces of the assembled viral core NEC [5,7,21,38,46,47,48]. At present, however, it is not clear whether the formation of oligomeric NEC structures is an initial event to facilitate the effector protein recruitment and viral capsid docking, or whether any initially associated effector protein may facilitate structural fine-modulation including extended NEC binding properties. Data of the present study indicate that formation of dimers, hexamers and higher oligomers may represent a strong intrinsic assembly property of the viral core NEC that may be significant for later steps of binding activities and regulation. Specifically, the NEC association of protein kinases, such as vCDK/pUL97, CDK1, CDK2, protein kinase C (PKC) and possibly even more, is another important characteristic shared between herpesviral NECs that leads to the phosphorylation-driven regulatory activities of the multicomponent extensions of these complexes [8,9,10,12,13,19,36,49]. Some of these events of site-specific phosphorylation of NEC-associated proteins have been functionally described, such as lamin A/C pSer22 phosphorylation [7,20,38], while others remain to be further investigated, such as the phosphorylation of core NEC proteins themselves, p32/gC1qR phosphorylation and the association of a variety of further phosphoproteins [4].

Thus, the study provides novel data of the characterization of the HCMV core NEC interaction properties, oligomerization and kinase association. In essence, the approach of intracellular as well as protein lysate-based chemical cross-linking demonstrated that NEC oligomerization is neither a crystallization artifact nor an activity resulting from overexpression, but should be considered as an intrinsic feature of the NEC formation in HCMV-infected cells. The antiviral activity identified for kinase inhibitors that exert NEC-directed inhibitory properties underlines the suitability of this antiviral strategy. Future investigations may clarify in detail the inhibitory efficacy of small molecules that interfere with core NEC hook-into-groove interaction, oligomeric NEC assembly or NEC-associated kinase activities.

## Supplementary Materials

The following are available online at https://www.mdpi.com/article/10.3390/v14051021/s1: Figure S1. In vitro assembly assay, performed under conditions of cross-linking and immunoprecipitation, to substantiate the association of CDK1 with oligomeric forms of the HCMV core NEC. Figure S2. Nuclear egress assay demonstrating the inhibitory activity of R25 and CDK2 Inh II.
