# Supplementary material for: The Oligomeric Assemblies of Cytomegalovirus Core Nuclear Egress Proteins Are Associated with Host Kinases and Show Sensitivity to Antiviral Kinase Inhibitors
Source: Viruses. 2022 May 11;14(5):1021. doi: 10.3390/v14051021 (PMC9146606; doi:10.3390/v14051021)
Supplement: Supplementary file 1 [file viruses-14-01021-s001.zip › viruses-1684349-supplementary.pdf]

viruses

MDPI

Supplementary Materials

The oligomeric assemblies of cytomegalovirus core nuclear egress proteins are associated with host kinases and show sensitivity to antiviral kinase inhibitors

Jintawee Kicuntod[1], Sigrun Häge[1], Friedrich Hahn[1], Heinrich Sticht[2] & Manfred Marschall[1,*]

[1] Institute for Clinical and Molecular Virology, Friedrich-Alexander-Universität Erlangen-Nürnberg (FAU), Erlangen, Germany; jintawee.kicuntod@extern.uk-erlangen.de (J.K.), sigrun.haege@fau.de (S.H.), friedrich.hahn@uk-erlangen.de (F.H.), manfred.marschall@fau.de (M.M.)

[2] Bioinformatics, Institute of Biochemistry, Friedrich-Alexander-Universität Erlangen-Nürnberg, Erlangen, Germany; heinrich.sticht@fau.de (H.S.)

* Correspondence: e-mail manfred.marschall@fau.de, phone +49 9131 8526089

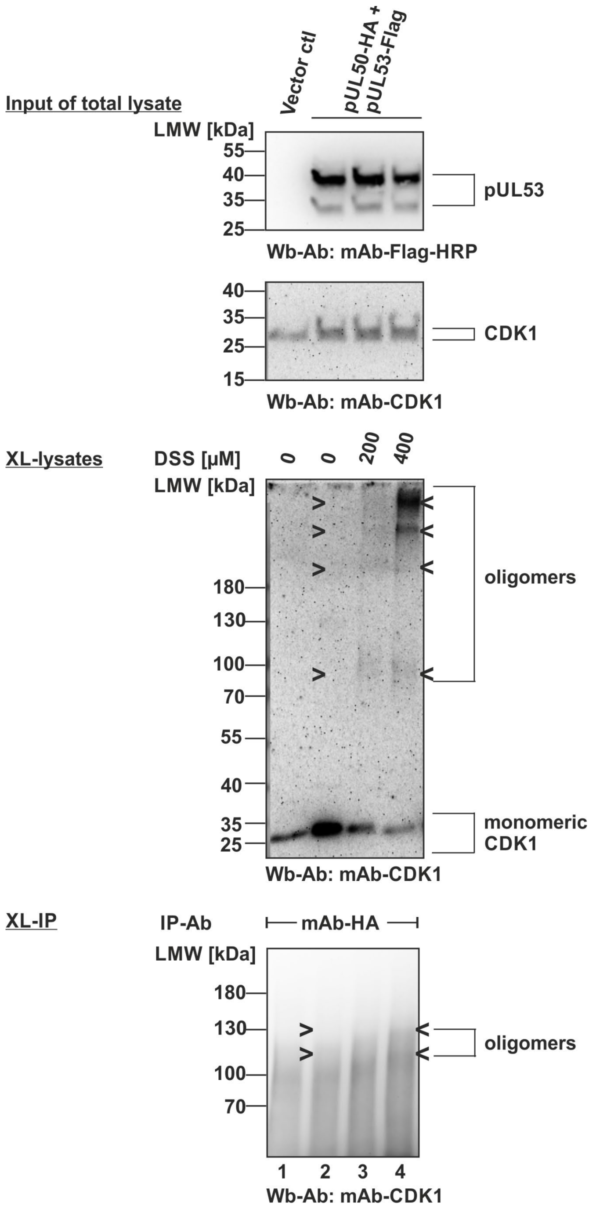

Figure S1. ***In vitro*** **assembly assay, performed under conditions of cross-linking and immunoprecipitation, to substantiate the association of CDK1 with oligomeric forms of the HCMV core NEC.** *In vitro* NEC assembly assay for chemical cross-linking was based on the single transient expression of tagged pUL50 and pUL53. Expression levels were monitored by Wb control stainings (input of total lysates). The two core NEC proteins were assembled in the presence of endogenous CDK1, before DSS was utilized to stabilize the complexes. Oligomer formation was demonstrated without (XL-lysates) or with immunoprecipitation (XL-IP), and Wb detection was achieved using tag-specific antibodies. LMW, low molecular weight marker.

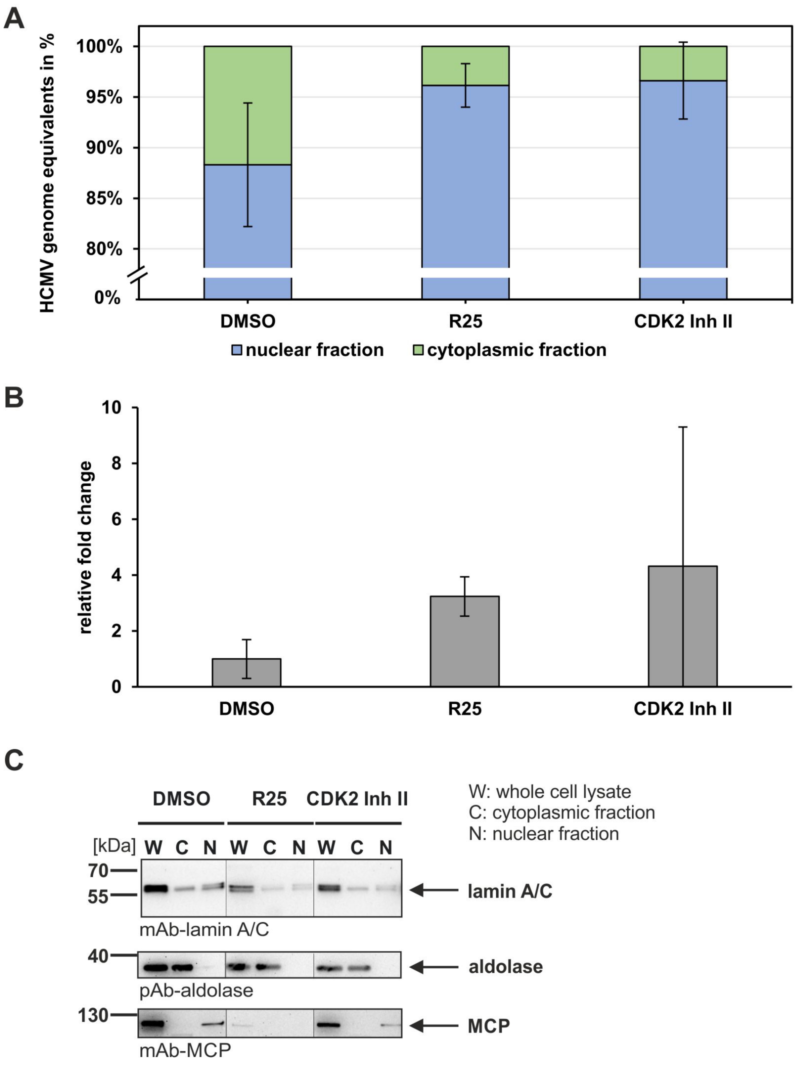

Figure S2. Nuclear egress assay demonstrating the inhibitory activity of R25 and CDK2 Inh II. HFF cells were infected with HCMV AD169 at a MOI of 0.01 and treated with 0.5 µM R25, 0.5 µM CDK2 Inh II or DMSO as a solvent control. At 6 d p.i., cells were harvested and the nuclear egress assay was performed. **(A)** Viral genomic loads were quantitated by qPCR and the results are illustrated as HCMV genome equivalents in %. **(B)** The fold change between the percentages of nuclear-to-cytoplasmic transition of viral genomes was calculated. Mean values ± SD are given. **(C)** Samples taken after fractionation were subjected to Wb analysis using the indicated antibodies. W, whole cell lysate; C, cytoplasmic fraction; N, nuclear fraction.
